# Improving Crystallization Properties, Thermal Stability, and Mechanical Properties of Poly(L-lactide)-*b*-poly(ethylene glycol)-*b*-poly(L-lactide) Bioplastic by Incorporating Cerium Lactate

**DOI:** 10.3390/polym16233367

**Published:** 2024-11-29

**Authors:** Arriya Chuangchai, Yodthong Baimark

**Affiliations:** Biodegradable Polymers Research Unit, Department of Chemistry and Centre of Excellence for Innovation in Chemistry, Faculty of Science, Mahasarakham University, Mahasarakham 44150, Thailand; 66010251008@msu.ac.th

**Keywords:** polylactide, triblock copolymer, cerium lactate, thermal properties, mechanical properties

## Abstract

The more flexible and faster biodegradation rate of poly(L-lactide)-*b*-poly(ethylene glycol)-*b*-poly(L-lactide) (PLLA-PEG-PLLA) triblock copolymer makes it a promising bioplastic compared to PLLA. However, finding effective additives for this triblock copolymer remains a research challenge for their wider applications. This work involved the melt-blending of a cerium lactate (Ce-LA) antibacterial agent with a triblock copolymer. The thermal properties, crystalline structures, mechanical properties, and phase morphology of the PLLA-PEG-PLLA/Ce-LA composites were examined. With 0.5 wt% Ce-LA, the composite exhibited the best crystallization properties. The crystallinity of the composite contained 0.5 wt% Ce-LA increased from 11.8 to 15.9%, and the half-time of crystallization decreased from 3.37 to 1.28 min at 120 °C, compared with the pure triblock copolymer. The incorporation of Ce-LA did not result in any changes to the crystalline structure of the triblock copolymer matrix. The best improvement in thermal stability and tensile properties of the composites was achieved with the addition of 1.5 wt% Ce-LA. When compared to the pure triblock copolymer, the temperature at maximum decomposition rate of PLLA blocks shifted from 310 °C to 327 °C, the tensile strength increased from 14.3 MPa to 19.5 MPa, and the Young’s modulus increased from 204 MPa to 312 MPa. This study concludes that the incorporation of Ce-LA enhanced the crystallizability, thermal stability, and mechanical properties of PLLA-PEG-PLLA, indicating that Ce-LA could serve as a versatile additive to the PLLA-PEG-PLLA bioplastic.

## 1. Introduction

The production and waste disposal of non-biodegradable commodity plastics, such as polyethylene, polypropylene, and polystyrene, increase greenhouse gas emissions and cause serious environmental problems [1,2]. They are fossil-based plastics. Replacing fossil-based plastics with biodegradable bio-based plastics, especially for single-use plastic packaging, can reduce greenhouse gas emissions, plastic waste pollution, and the carbon footprint [1,2,3,4,5]. Poly(L-lactide) (PLLA) has attracted much attention because of its biorenewability, biocompatibility, biodegradability, biocompostability, and the ease of melt processing it [6,7,8,9,10,11]. Hydrolysis can degrade PLLA into small molecules, such as oligomers, dimers, and monomers. Enzymes in human and animal bodies and microorganisms, such as bacteria and fungi, in compost degrade these small molecules into carbon dioxide and water. The biodegradation rate of PLLA in human and animal bodies was faster than in soil burial [12]. However, high brittleness and long-term biodegradation of PLLA limit its packaging applications [13,14,15,16].

Triblock copolymers of PLLA-*b*-poly(ethylene glycol)-*b*-PLLA (PLLA-PEG-PLLA) with high molecular weight showed promise for use in flexible bioplastic applications because they were more flexible and had faster biodegradation than PLLA [17,18,19]. The hydrophilic PEG blocks induced a plasticization effect [19]. However, some properties of PLLA-PEG-PLLA, such as crystallization and mechanical properties, remain to be improved for wider applications. To achieve this, we have blended PLLA-PEG-PLLA with various fillers, including calcium carbonate [20], zinc oxide nanoparticles [21], high-density polyethylene [22], and biochar [23]. These fillers acted as multi-functional fillers for PLLA-PEG-PLLA to enhance the crystallizability [20,21,22,23], thermal stability [20,22,23], mechanical properties [20,21,23], and antibacterial properties [21]. However, research on effective fillers and additives for PLLA-PEG-PLLA remains a challenge for its development in wider applications.

A cerium lactate (Ce-LA) rare earth complex, synthesized from rare earth cerium and lactic acid ligand, has been blended with PLLA to improve certain properties [24]. Ce-LA enhanced the nucleating effect and antibacterial properties of PLLA, but its incorporation resulted in a reduction in PLLA’s thermal stability. However, no studies have examined the effectiveness of the Ce-LA in enhancing various properties of PLLA-PEG-PLLA. In this work, we prepared PLLA-PEG-PLLA/Ce-LA composites by melt mixing. The influence of Ce-LA on the crystallizability, crystalline structures, thermal stability, phase morphology, and mechanical properties of the composites was determined.

## 2. Materials and Methods

### 2.1. Materials

The PLLA-PEG-PLLA triblock copolymer, with a number-averaged molecular weight (*M_n_*) of 108,500 and a dispersity (*Ð*) of 2.2, determined by gel permeation chromatography (GPC), was synthesized via the ring-opening polymerization of the L-lactide monomer with a chain extender, as detailed in our prior studies [20,21]. The synthesis reaction of the triblock copolymer is presented in Figure S1. The PEG (M.W. of 20,000) and stannous octoate (95%) obtained from Sigma-Aldrich (Burlington, MA, USA) were chosen as the initiating system. Joncryl^TM^ ADR 4368 chain extender was purchased from BASF (Bangkok, Thailand). Cerium (III) nitrate hexahydrate (CeN_3_O_9_·6H_2_O, 99.5%, Acors Organics, Geel, Belgium) and sodium lactate solution in water (C_3_H_5_NaO_3_, 60 wt%, Thermo Scientific, Waltham, MA, USA) were used to prepare the cerium lactate (Ce-LA).

### 2.2. Preparation of Cerium Lactate (Ce-LA)

**Figure 1:**
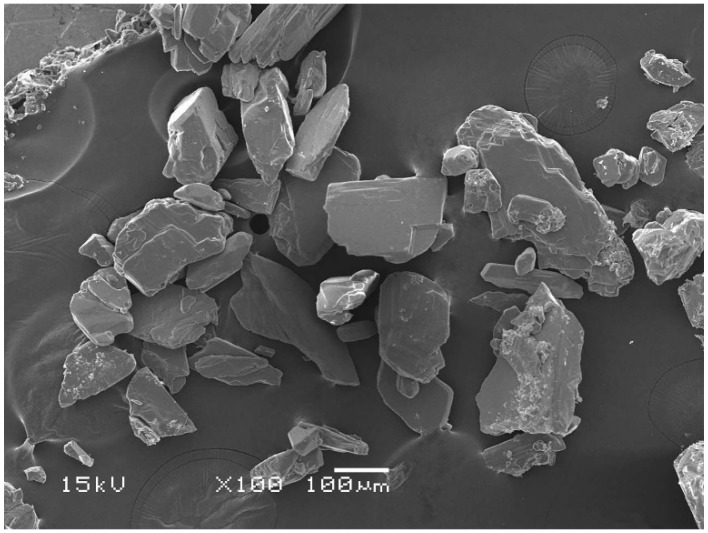
SEM micrograph of Ce-LA powder.

**Figure 2:**
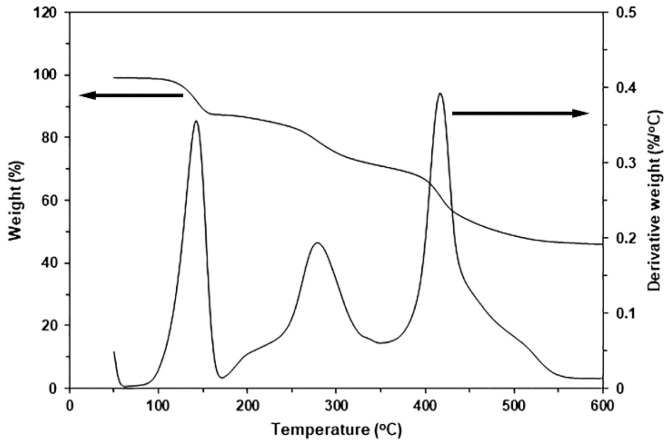
TG and DTG thermograms of Ce-LA powder.

Ce-LA was prepared from cerium (III) nitrate hexahydrate and a sodium lactate solution in water as described in the literature [24]. The synthesis reaction of Ce-LA is illustrated in Figure S2. A mixed solution of cerium (III) nitrate and sodium lactate in deionized water, with a 1:3 molar ratio, was stirred at 35 °C for 4 h. The mixed solution was then adjusted to pH 7 with ammonia before being allowed to precipitate for 12 h. The resulting Ce-LA powder was collected by centrifugation and then vacuum-dried at 100 °C for 12 h. A SEM micrograph of the Ce-LA powder is illustrated in Figure 1. Figure 2 illustrates the TG and DTG thermograms of the Ce-LA powder. It shows three decomposition steps of the Ce-LA, which consisted of a water evaporation stage at 100–150 °C, a breaking stage of the cerium-lactic acid coordination bonds at 200–350 °C, and a lactic acid decomposition stage. At 600 °C, the residue weight was approximately 45%, primarily due to the presence of CeO_2_ [24].

### 2.3. Preparation of PLLA-PEG-PLLA/Ce-LA Composites

The triblock copolymer and Ce-LA were vacuum-dried for 24 h at 50 °C. PLLA-PEG-PLLA/Ce-LA composites were then produced by a melt-mixing technique at 190 °C for 8 min using a torque rheometer (Polylab OS System model, HAAKE, Waltham, MA, USA) with a rotor speed of 100 rpm. Composites with Ce-LA contents of 0.5, 1.0, 1.5, 2.0, and 2.5 wt% were prepared. The composite films were prepared by a hot-press technique using a hot-press machine (Auto CH model, Carver, Wabash, IN, USA) under 5 MPa force at 180 °C for 2 min before cooling to room temperature with water cooling plates under the same conditions. The film thicknesses were 0.2–0.3 mm.

### 2.4. Characterization of PLLA-PEG-PLLA/Ce-LA Composites

A differential scanning calorimeter (DSC, Pyris Diamond model, PerkinElmer, Waltham, MA, USA) was used to determine the thermal transition characteristics of the samples. For the DSC heating scan, the sample’s thermal history was removed by melting it at 200 °C for 3 min and then cooling it to 0 °C at a rate of 100 °C/min before heating it to 200 °C at a rate of 10 °C/min. The sample was then held at 200 °C for 3 min before cooling to 0 °C at a rate of 10 °C/min for the DSC cooling scan. The degree of crystallinity (*X_c_*) was calculated using Equation (1) derived from the DSC heating thermograms.

*X_c_* (%) = [(Δ*H_m_* − Δ*H_cc_*)/(93.6 × *W_PLLA_*)] × 100 (1)

where the enthalpies of melting and cold crystallization are denoted by the symbols ∆*H_m_* and ∆*H_cc_*, respectively. The 93.6 J/g is a Δ*H_m_* of 100% *X_c_* PLLA [20]. *W_PLLA_* is the weight fraction of PLLA in the composites.

For DSC isothermal scans, the sample’s thermal history was erased by melting it at 200 °C for 3 min, followed by cooling it to 120 °C at a rate of 50 °C/min. They were then isothermally crystallized at 120 °C until the exothermic crystallization process was completed, in accordance with the literature [25].

X-Ray diffraction was used to determine the sample’s crystalline structures using an X-Ray diffractometer (XRD, D8 Advance model, Bruker Corporation, Karlsruhe, Germany) with CuKα radiation operating at 40 kV and 40 mA. The step size was 0.02°, and the scan speed was 0.02 s/step.

The fractured surfaces of the samples were observed using a scanning electron microscope (SEM, JSM-6460LV model, JEOL, Tokyo, Japan). The film samples were cryogenically fractured with liquid nitrogen and deposited with gold before being scanned at 20 kV.

The thermal stability of the samples was investigated using a thermogravimetric analyzer (TGA, SDT Q600 model, TA Instrument, New Castle, DE, USA) under a nitrogen flow of 100 mL/min with a 20 °C/min heating rate.

The tensile properties of the samples were measured using a universal testing machine (LY-1066B model, Dongguan Liyi Environmental Technology Co., Ltd., Dongguan, China) at 25 °C with a 100 kg load cell. Uniaxial tensile tests were carried out on rectangular-shaped films with a 50 mm/min constant deformation rate and a 50 mm distance between the fixtures. The results of tensile properties correspond to the average of at least five measurements.

## 3. Results and Discussion

### 3.1. Thermal Transition Properties

**Figure 3:**
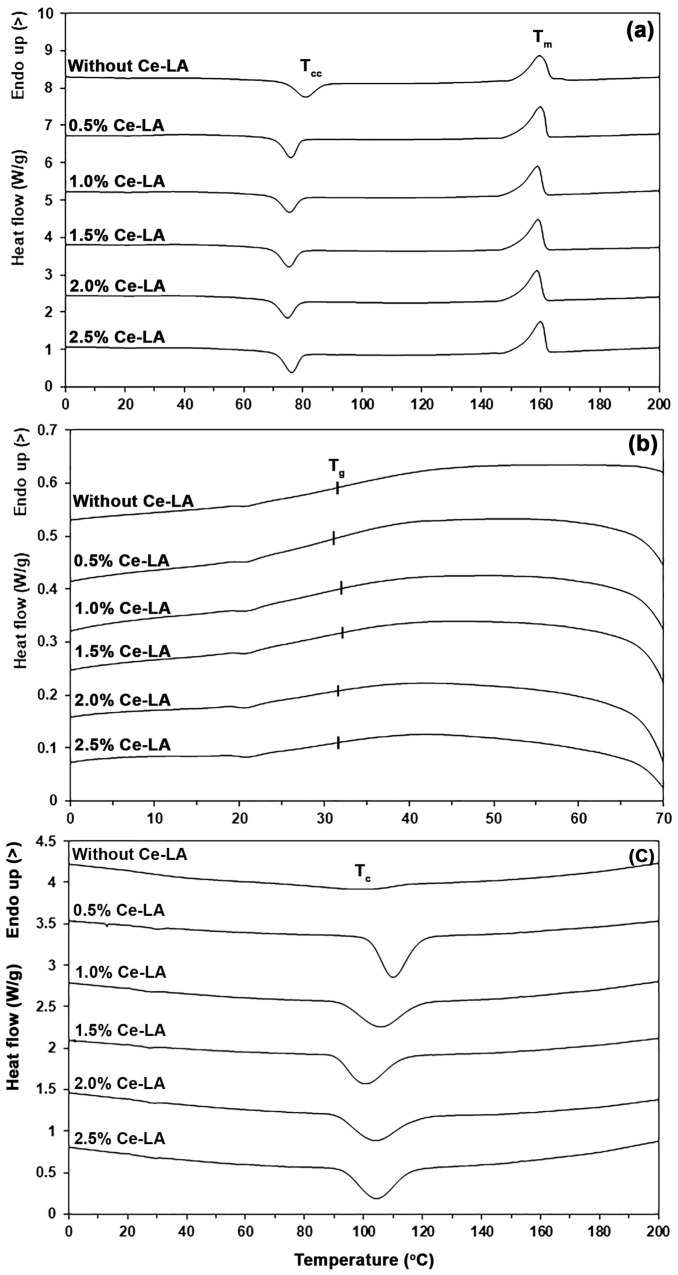
(**a**) DSC heating thermograms, (**b**) expanded *T_g_* regions, and (**c**) DSC cooling thermograms of PLLA-PEG-PLLA/Ce-LA composites.

The DSC heating and cooling thermograms were used to determine the thermal transition characteristics and are presented in Figure 3. The DSC heating and cooling thermograms of the Ce-LA had no DSC peak, as shown in Figure S3. Table 1 provides a summary of the results from the DSC heating and cooling thermograms. Figure 3b illustrates the expanded thermograms of the glass transition temperature (*T_g_*) regions. The *T_g_* and melting temperatures (*T_m_*) of the pure triblock copolymer were 32 °C and 159 °C, respectively. The *T_g_* of PLLA-PEG-PLLA was lower than that of the PLLA (approximately 60 °C) because the plasticizing effect was induced with the PEG blocks [17,18]. The *T_g_* and *T_m_* values of all the composites ranged from 31 to 32 °C and 159 to 160 °C, respectively, suggesting that the addition of Ce-LA did not affect both the transition of glassy-to-rubbery state and melting behavior of the triblock copolymer. It was found that the incorporation of Ce-LA shifted the cold crystallization temperature (*T_cc_*) peaks from 81 °C to lower temperatures (75–76 °C), suggesting an improvement in the crystallization of polymer matrices [26,27,28]. Thus, this result suggests that the addition of Ce-LA enhanced the crystallization of the triblock copolymer matrix. Table 1 also reports the degree of crystallinity (*X_c_*) values. The *X_c_* values increased from 11.8% to 15.9% with the addition of 0.5 wt% Ce-LA, which supports the idea that Ce-LA enhances the crystallizability of triblock copolymer by enhancing the heterogeneous nucleating effect. The *X_c_* value decreased again when the Ce-LA content was higher than the 0.5 wt%. This may be due to the aggregation of the inorganic fillers at higher loading in the polymeric matrix, suppressing its nucleation effectiveness [20,21,29]. However, the *X_c_* values of all the composites in the range of 13.7–15.9% were still higher than that of the pure triblock copolymer (11.8%).

In Figure 3c, exothermic peaks of crystallization temperature (*T_c_*) were detected, and Table 1 also provides the *T_c_* values. The *T_c_* peak of the pure triblock copolymer (99 °C) shifted to a higher temperature (110 °C) with the incorporation of 0.5 wt% Ce-LA, enhancing crystallization of the triblock copolymer. The crystallization rate of the triblock copolymer matrix was accelerated by the heterogeneous nucleating agents, as indicated by the *T_c_* peak shifting to a higher temperature during the DSC cooling scan [26,30,31,32]. The *T_c_* peak shifted to a lower temperature again when the Ce-LA content was over 0.5 wt%. However, the *T_c_* values of all the composites (102–110 °C) were still higher than that of the pure triblock copolymer (99 °C), indicating that the crystallizability of all the composites was better than that of the pure triblock copolymer.

**Figure 4:**
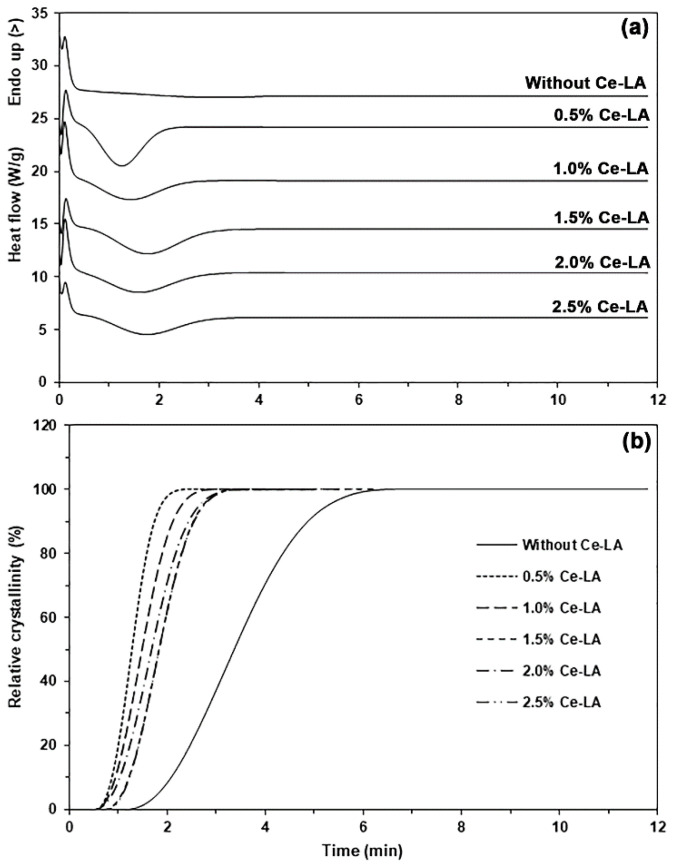
(**a**) Isothermal crystallization thermograms and (**b**) curves of relative crystallinity-time of PLLA-PEG-PLLA/Ce-LA composites.

Additionally, we examined the crystallization characteristics of the composites using the half-time of crystallization (*t*_1/2_), which was obtained from Figure 4. The polymer samples exhibited a relative crystallinity of 50% during isothermal scans at *t*_1/2_, as shown in Figure 4b. The obtained *t*_1/2_ values are reported in Table 2. The *t*_1/2_ values decreased from 3.4 min to 1.3 min with the addition of 0.5 wt% Ce-LA, which confirmed that the triblock copolymer matrix was heterogeneously nucleated by the Ce-LA. The *t*_1/2_ value increased again when the Ce-LA content was higher than 0.5 wt%. Aggregation of Ce-LA particles may reduce nucleation effectiveness. However, the *t*_1/2_ values of all the composites (1.3–1.8 min) were still higher than that of the pure triblock copolymer (3.4 min), indicating that all the composites had a faster crystallization rate than that of the pure triblock copolymer. The following Avrami Equation (2) was employed to analyze the crystallization kinetics of composite materials through the Avrami exponent (*n*) and the crystallization rate constant (*k*) [25,33].

1 − *X_t_* = exp(−*kt^n^*) (2)

where *X_t_* is the accumulated relative crystallinity, and *t* is the time for crystallization.

The *n* and *k* values obtained from log[−ln(1 − *X_t_*)] versus log(*t*) graphs are also summarized in Table 2. All *R*^2^ values exceeded 0.99, indicating that the graphs exhibited excellent linear regression. The pure triblock copolymer had higher *n* and lower *k* values compared to the composites, indicating that it has the slowest crystallization [33,34]. The composite containing 0.5 wt% Ce-LA had a lower *n* value and higher *k* value compared to the pure triblock copolymer, suggesting that added Ce-LA accelerated the triblock copolymer’s crystallization. The *n* value increased and the *k* value decreased when the Ce-LA content was over 0.5 wt%. The aggregation of Ce-LA may have reduced nucleation efficiency. The results will be confirmed later using SEM analysis. However, the *n* values of all the composites (3.3191–3.5364) were still lower and the *k* values were still higher (0.0862–0.3590 min^−1^) than those of the pure triblock copolymer (*n* = 3.5916 and *k* = 0.0086 min^−1^). This confirmed that all the composites had a faster crystallization rate than that of the pure triblock copolymer. All the *n* values exceeded 2.0, indicating a heterogeneous nucleating effect [35]. It is clear from the DSC results that Ce-LA is a good nucleating agent for triblock copolymer.

### 3.2. Crystalline Structures

**Figure 5:**
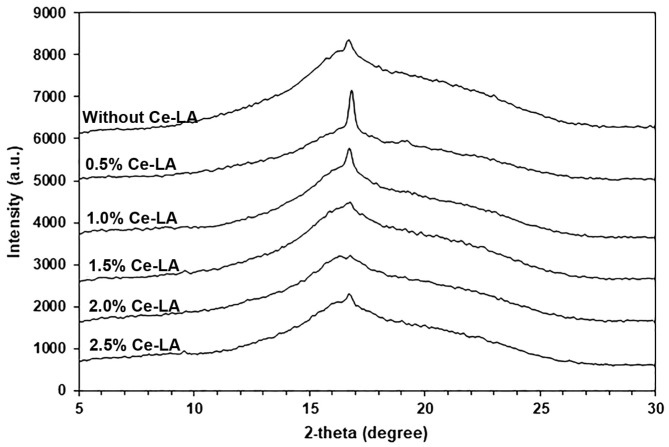
XRD profiles of PLLA-PEG-PLLA/Ce-LA composites.

X-Ray diffraction was used to evaluate the crystalline structures of the samples. As shown in Figure 5, the pure triblock copolymer exhibited a broad diffraction peak at 16.9° (200)/(110), which is attributed to the crystallites of the PLLA blocks [32,36,37]. All the composites also exhibited an XRD peak at 16.9° corresponding to the *α* crystalline phase with an orthorhombic unit cell of PLLA [38,39], indicating that the addition of Ce-LA did not alter the crystalline structure of the triblock copolymer matrix. An XRD profile of Ce-LA exhibited diffraction peaks at 10.4° (011), 15.2° (200), 17.8° (102), and 22.2° (212), as shown in Figure S4, attributed to Ce-LA’s crystallites [24]. The XRD profiles of the composites had no XRD peaks of Ce-LA. This may be because they were overlapped by the amorphous halo of the triblock copolymer matrix. It can be clearly seen that the peak intensity at 16.9° increased when the 0.5 wt% Ce-LA was included, suggesting that the triblock copolymer’s crystallinity increased. This supports the idea that Ce-LA enhances the nucleating effect. However, the peak intensity decreased again when the Ce-LA content exceeded 0.5 wt%. This may be explained by the Ce-LA being aggregated when the amount of Ce-LA was higher than 0.5 wt%, decreasing its nucleation effectiveness, as described in the later SEM analysis.

### 3.3. Phase Morphology

**Figure 6:**
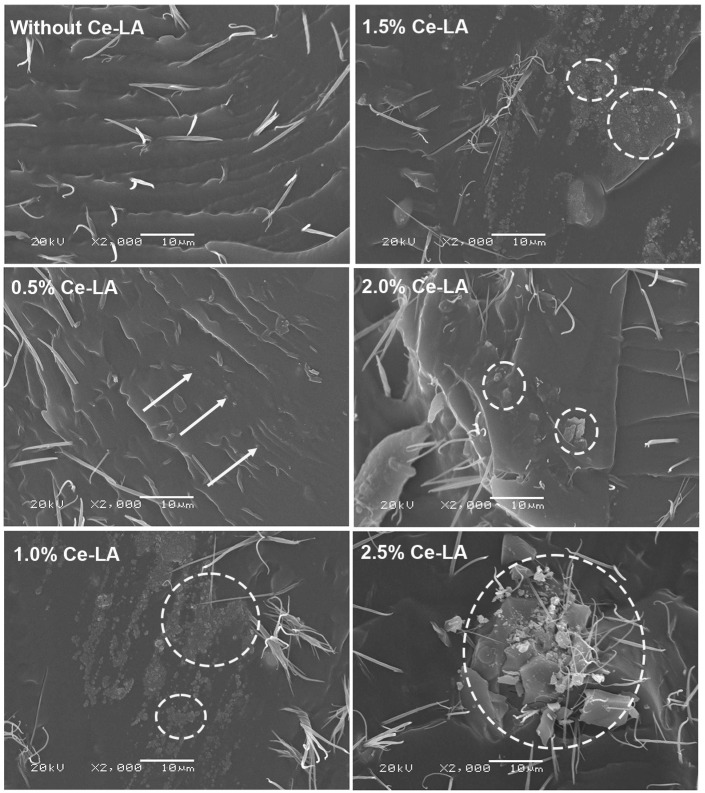
SEM micrographs of film cross-sections of PLLA-PEG-PLLA/Ce-LA composite films (some Ce-LA particles were labeled by white arrows, and some Ce-LA aggregates were labeled by white circles).

Figure 6 illustrates the SEM micrographs of film cross-sections of the film samples. The film cross-section of the pure triblock copolymer exhibited rough surfaces due to its high flexibility. Additionally, we observed the presence of tiny needles on the film cross-section. The high flexibility of triblock copolymer matrix may have caused it to stretch prior to film fracture. All of the composite films also have tiny needles on film cross-sections, indicating that they are flexible.

The Ce-LA particles were well distributed on the film matrix for 0.5 wt% Ce-LA. However, aggregation of small Ce-LA particles was observed when the Ce-LA content was over 0.5 wt%. This may be due to the difference in hydrophilicity between the polymer matrix and the inorganic fillers [40,41,42]. The composites contained smaller Ce-LA particles than the originals (see Figure 1). This may be due to the breakdown of Ce-LA particles during the melt-mixing process. The SEM results could explain the decrease in nucleation effectiveness of Ce-LA as its content increased beyond 0.5 wt%, as described in the above DSC and XRD analyses. Aggregation of large Ce-LA particles was found for the 2.0 and 2.5 wt% Ce-LA composites. The high Ce-LA content made particles difficult to break during the melt-mixing process. However, good wettability between the triblock copolymer matrix and the Ce-LA was found. The Ce-LA particles did not fall out from the triblock copolymer matrix during cryofracture.

### 3.4. Thermal Decomposition Characteristics

**Figure 7:**
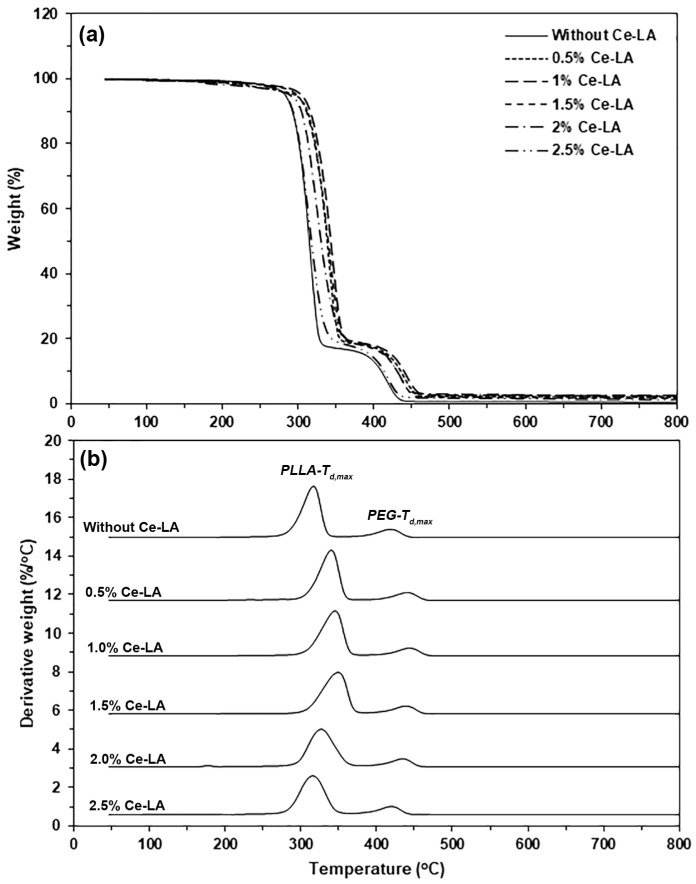
(**a**) TG and (**b**) DTG thermograms of PLLA-PEG-PLLA/Ce-LA composites.

The TG and DTG thermograms (Figure 7a and Figure 7b, respectively) were used to investigate thermal decomposition characteristics. Table 3 summarizes the TG and DTG results. As shown in Figure 7a, the pure triblock copolymer thermally decomposed in two steps at different temperatures: PLLA blocks decomposed first, at 250–350 °C, and PEG blocks decomposed at 350–450 °C [23,43]. The thermal decomposition ranges of the PLLA blocks shifted to higher temperatures with the incorporation of Ce-LA, but those of the PEG blocks did not shift, showing that adding Ce-LA improved the PLLA block’s thermal stability. The DTG thermogram analysis will confirm this phenomenon. As shown in Table 3, the residue weights of the composites at 600 °C (0.75–2.12%) were higher than that of the pure triblock copolymer (0.39%) and consistently increased with Ce-LA content. This is because Ce-LA has a high residue weight at 600 °C (45%), as shown in Figure 2. The resulting residue weights of the composites in Table 3 were higher than the predicted values, which were calculated based on residue weights at 600 °C of pure triblock copolymer (0.39%) and Ce-LA (45%). This may be explained by the adhesion of some char of the decomposed triblock copolymer over the Ce-LA surfaces, which induced excess residue weight at 600 °C of the composites.

The temperature at maximum decomposition rate (*T_d_*_,*max*_) peaks of the PLLA blocks (*PLLA*-*T_d_*_,*max*_) and the PEG blocks (*PEG*-*T_d_*_,*max*_) were detected in the DTG thermograms (Figure 7b). Table 3 summarizes these *T_d_*_,*max*_ values. The pure triblock copolymer had *PLLA*-*T_d_*_,*max*_ and *PEG-T_d.max_* peaks at 310 °C and 418 °C, respectively, while the composites had *PLLA*-*T_d_*_,*max*_ values between 311 °C and 327 °C, supporting the idea that the incorporation of Ce-LA improved the PLLA block’s thermal stability. The *PLLA*-*T_d_*_,*max*_ value increased with Ce-LA content until 1.5 wt%. This may be due to the large Ce-LA particles being aggregated when the Ce-LA content was higher than 1.5 wt%, as described in the above SEM analysis. The *PLLA*-*T_d_*_,*max*_ value then decreased again when the Ce-LA content exceeded 1.5 wt%. The *PEG*-*T_d_*_,*max*_ values of composites (417–419 °C) were nearly equal to that of the pure triblock copolymer (418 °C).

According to the report, the incorporation of Ce-LA reduced the intermolecular forces between the PLLA chains, thereby decreasing the PLLA’s thermal stability [24]. Surprisingly, this work found that the added Ce-LA enhanced the thermal stability of the PLLA blocks. We have reported that blending triblock copolymer with inorganic fillers, such as calcium carbonate (CaCO_3_) [20] and zinc phenylphosphate (PPZn) [44], improved the PLLA block’s thermal stability. The thermal stability of the triblock copolymer matrix was improved by CaCO_3_, but the PLLA matrix did not experience any improvement. Therefore, the improved phase compatibility between the PLLA blocks and Ce-LA, facilitated by the PEG blocks, may explain the enhanced heat transfer between them and the resulting increase in thermal stability.

### 3.5. Tensile Properties

**Figure 8:**
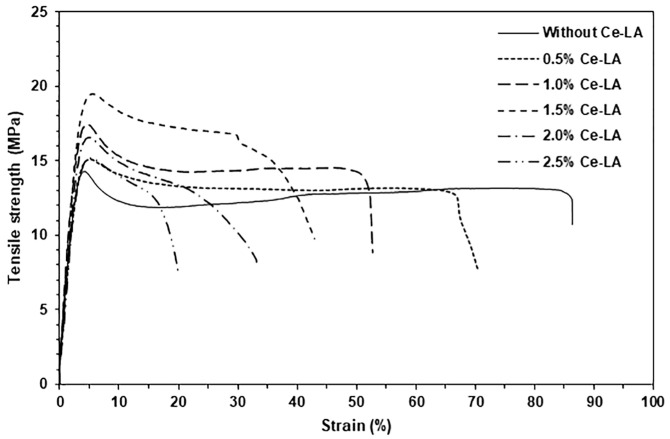
Tensile curves of PLLA-PEG-PLLA/Ce-LA composites.

Figure 8 displays the tensile curves of the sample films, while Table 4 summarizes the results of the tensile test. According to the above SEM analysis, the tensile curve of the pure triblock copolymer showed a yield point, suggesting that it had high flexibility. The incorporation of Ce-LA increased both the tensile strength and Young’s modulus of films until the Ce-LA content was 1.5 wt%. The ultimate tensile strength of the composite film increased by 36%, and the Young’s modulus increased by 53% when 1.5% Ce-LA was incorporated, compared to the pure triblock copolymer film. The triblock copolymer’s mechanical properties were improved by the Ce-LA particles’ fine dispersion within the matrix [28,42,45]. These tensile values decreased again when the Ce-LA content was over 1.5 wt%. This may be explained by the decrease in the reinforcing effectiveness of Ce-LA, which could occur from the aggregation of large Ce-LA particles, as detailed in the above SEM results, which reduce stress transfer between polymer matrices and filler particles [46,47].

All of the composites exhibited ultimate tensile strength and Young’s modulus values that were greater than those of the pure triblock copolymer. This suggests that Ce-LA enhanced the reinforcing effect for the triblock copolymer. Additionally, adding Ce-LA to increase the crystallinity of the triblock copolymer (see Table 1) likely also enhanced the ultimate tensile strength and Young’s modulus of the film samples. This is because the crystallites of the triblock copolymer matrix acted as physical cross-linked sites. As the Ce-LA content increased, the strain at break in the composites steadily decreased. It is well known that the incorporation of inorganic fillers decreases the flexibility of polymeric matrices because the rigid fillers restrict the ductile flow of polymer chains [41,48]. All the composites showed a yield point, confirming the above SEM analysis’s conclusion that all of the composites remained flexible.

## 4. Conclusions

In summary, the PLLA-PEG-PLLA/Ce-LA composites were prepared via melt-blending with Ce-LA content ranging from 0.5 to 2.5 wt%. The crystallization properties, crystalline structures, phase morphology, thermal stability, and tensile properties of the composites were investigated in detail. The results indicated that the 0.5 wt% Ce-LA exhibited the best nucleation efficiency for the triblock copolymer matrix. The highest *X_c_*, the highest *T_c_*, and the lowest *t*_1/2_ values of the composites were obtained from DSC analysis when 0.5 wt% Ce-LA was incorporated. The 1.5 wt% Ce-LA composite had the best thermal stability and tensile properties of the composites. The highest *PLLA*-*T_d_*_,*max*_ and the highest tensile strength values of the composites were obtained from TGA and tensile analyses, respectively, when the Ce-LA content was 1.5 wt%. From SEM analysis, aggregation of small Ce-LA particles was found when the Ce-LA content was higher than 0.5 wt%, whereas aggregation of large Ce-LA particles was found when the Ce-LA content exceeded 1.5 wt%. We can conclude that Ce-LA served as an effective nucleating agent, thermal stabilizer, and reinforcing agent for the triblock copolymer. The PLLA-PEG-PLLA/Ce-LA composites may be used as flexible food packaging materials. Future research should focus on the barrier, biodegradation, and antibacterial properties of these composites, which are crucial characteristics of biodegradable food packaging materials.

## Figures and Tables

**Table 1:** DSC results of PLLA-PEG-PLLA/Ce-LA composites from DSC heating and cooling thermograms.

Ce-LA Content (wt%)	*T_g_* (°C) ^a^	*T_cc_* (°C) ^a^	Δ*H_cc_* (J/g) ^a^	*T_m_* (°C) ^a^	Δ*H_m_* (J/g) ^a^	*X_c_* (%) ^a^	*T_c_* (°C) ^b^
-	32	81	20.3	159	31.3	11.8	99
0.5	31	75	17.6	160	32.4	15.9	110
1.0	32	75	16.0	159	29.7	14.8	106
1.5	32	75	17.4	159	30.6	14.3	102
2.0	31	75	17.8	159	30.6	14.1	103
2.5	31	76	17.6	160	30.1	13.7	103

^a^ Obtained from DSC heating thermograms. ^b^ Obtained from DSC cooling thermograms.

**Table 2:** DSC results of PLLA-PEG-PLLA/Ce-LA composites from isothermal crystallization thermograms.

Ce-LA Content (wt%)	*t*_1/2_ (min)	*n*	*k* (min^−1^)	*R^2^*
-	3.4	3.5916	0.0086	0.9962
0.5	1.3	3.3191	0.3590	0.9999
1.0	1.5	3.4340	0.1786	0.9997
1.5	1.8	3.5364	0.0862	0.9999
2.0	1.7	3.4335	0.1152	0.9988
2.5	1.8	3.4388	0.0919	0.9999

**Table 3:** TG and DTG results of PLLA-PEG-PLLA/Ce-LA composites.

Ce-LA Content (wt%)	Residue Weight at 600 °C (%) ^a^	*PLLA*-*T_d_*_,*max*_ (°C) ^b^	*PEG*-*T_d_*_,*max*_ (°C) ^b^
-	0.39	310	418
0.5	0.75	316	417
1	1.06	319	419
1.5	1.47	327	417
2	1.88	313	418
2.5	2.12	311	417

^a^ Obtained from Figure 7a. ^b^ Obtained from Figure 7b.

**Table 4:** Tensile properties of PLLA-PEG-PLLA/Ce-LA composites.

Ce-LA Content (wt%)	Ultimate Tensile Strength (MPa)	Strain at Break (%)	Young’s Modulus (MPa)
-	14.3 ± 0.6	86.3 ± 2.4	204 ± 18
0.5	15.1 ± 0.4	70.5 ± 5.1	230 ± 20
1	17.4 ± 0.6	52.6 ± 6.5	258 ± 15
1.5	19.5 ± 0.5	42.9 ± 5.2	312 ± 21
2	16.6 ± 0.3	33.2 ± 4.7	262 ± 24
2.5	15.2 ± 0.4	19.9 ± 6.8	254 ± 14

## Data Availability

Data are contained within the article.

## Supplementary Materials

The following supporting information can be downloaded at: https://www.mdpi.com/article/10.3390/polym16233367/s1, Figure S1: Synthesis reaction of chain-extended PLLA-PEG-PLLA; Figure S2: Synthesis reaction of Ce-LA; Figure S3: DSC heating and cooling thermograms of Ce-LA; Figure S4: XRD profile of Ce-LA.
