# Supplementary material for: Improving Crystallization Properties, Thermal Stability, and Mechanical Properties of Poly(L-lactide)-b-poly(ethylene glycol)-b-poly(L-lactide) Bioplastic by Incorporating Cerium Lactate
Source: Polymers (Basel). 2024 Nov 29;16(23):3367. doi: 10.3390/polym16233367 (PMC11644345; doi:10.3390/polym16233367)
Supplement: Supplementary file 1 [file polymers-16-03367-s001.zip › polymers-3278964-supplementary.pdf]

polymers

MDPI

Supplementary Materials

Improving Crystallization Properties, Thermal Stability, and Mechanical Properties of Poly(L-lactide)-*b*-poly(ethylene glycol)-*b*-poly(L-lactide) Bioplastic by Incorporating Cerium Lactate

Arriya Chuangchai and Yodthong Baimark *

Biodegradable Polymers Research Unit, Department of Chemistry and Centre of Excellence for Innovation in Chemistry, Faculty of Science, Mahasarakham University, Mahasarakham, 44150, Thailand; 66010251008@msu.ac.th
* Correspondence: yodthong.b@msu.ac.th

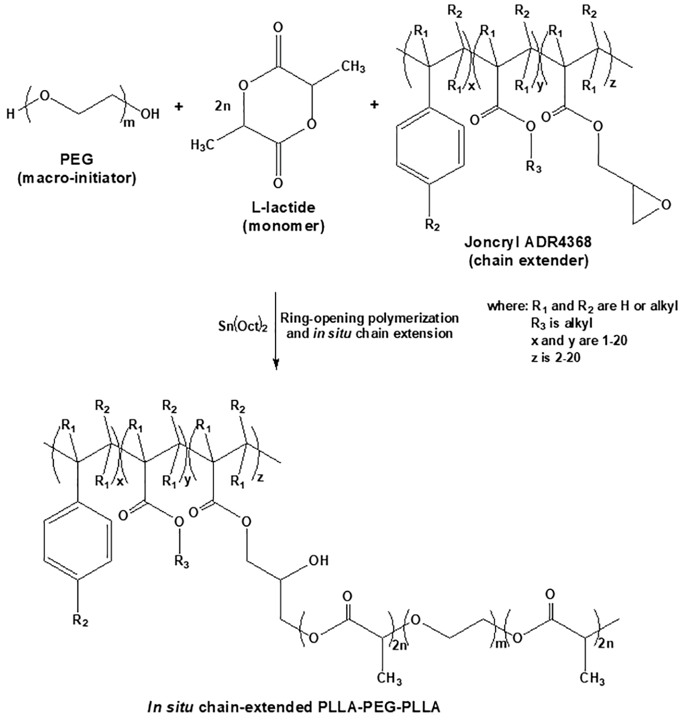

Figure S1 Synthesis reaction of chain-extended PLLA-PEG-PLLA.

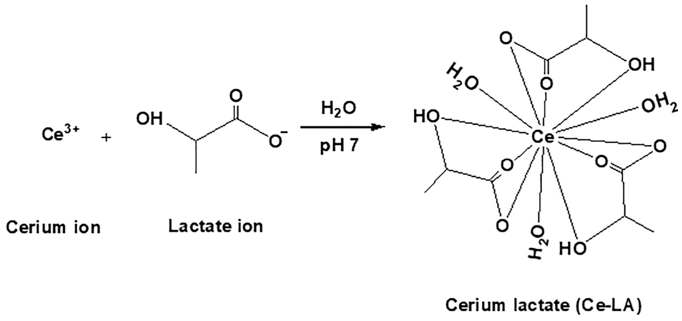

Figure S2 Synthesis reaction of Ce-LA.

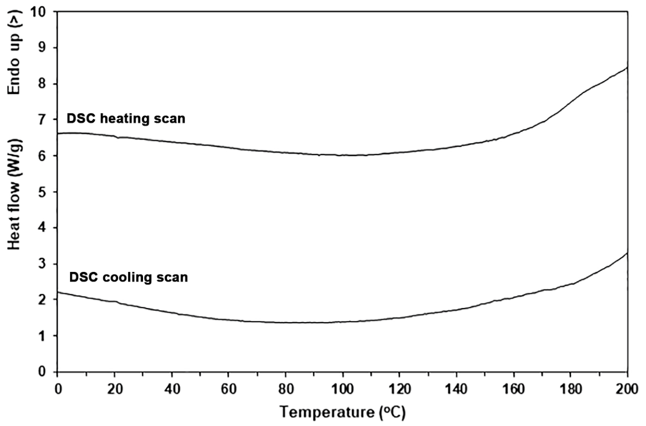

Figure S3 DSC heating and cooling thermograms of Ce-LA.

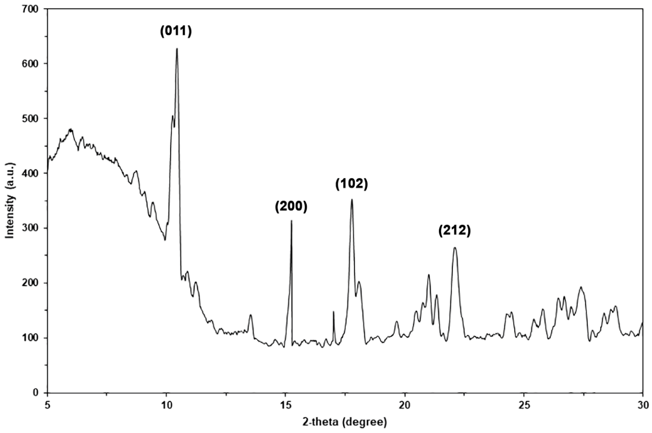

Figure S4 XRD profile of Ce-LA.
